# Density-dependent interspecific interactions and the complexity–stability relationship

**DOI:** 10.1098/rspb.2018.0698

**Published:** 2018-05-23

**Authors:** Kazutaka Kawatsu, Michio Kondoh

**Affiliations:** Graduate School of Life Sciences, Tohoku University, 6-3, Aoba, Aramaki, Aoba-ku, Sendai 980-8578, Japan

**Keywords:** coexistence, competition, food-web, functional response, interaction-type diversity, mutualism

## Abstract

Ever since May theorized that communities with larger numbers of species or interspecific interactions are inherently unstable, the mechanism allowing for the stable existence of complex communities in nature has been a central question in ecology. The main efforts to answer this question have sought to identify non-random features of ecological systems that can reverse a negative complexity–stability relationship into a positive one, but are far from successful, especially in their generality. Here, using the traditional community matrix analysis, we show that variation in the density dependence of interspecific interactions, which should be ubiquitous in nature, can dramatically affect the complexity–stability relationship. More specifically, we reveal that a positive complexity–stability relationship arises when harmful interspecific effects have larger density dependence than beneficial ones, regardless of the signs (i.e. positive or negative) of their dependence. Furthermore, numerical simulations demonstrated the synergistic stabilizing effect of interaction type diversity and density-dependence variation. Thus, this concept of density-dependence variation advances our understanding of the complexity–stability relationship in the real world.

## 1. Introduction

In natural ecological communities, a number of species are connected to each other via diverse ecological relationships, forming a complex network of interspecific interactions. In earlier times, this ecological complexity (i.e. species diversity and dense interactions) was expected to stabilize community dynamics (e.g. [1,2]). However, in the 1970s, this view was opposed in a theoretical study by May, which demonstrated with a mathematical model that a large, complex community is unlikely to be stable against even subtle perturbations [3]. Since May's seminal paper, the ‘complexity–stability’ issue has been one of the most highly debated topics in community ecology, and a number of researchers have been motivated to seek biological and ecological mechanisms that contribute to the stability of complex communities (reviewed in [4–6]).

Density dependence is a key feature that characterizes interspecific effects. An interspecific effect is defined as the magnitude of change in recipient population density caused by a donor species. According to ecological theory, an interspecific effect can be separated into two parts: a numerical response and a functional response. The former is measured by the changes in density of the recipient species caused by slight changes in the density of the donor species (e.g. considering the benefits of a predator–prey interaction, the recipient and the donor species are the predator and the prey, respectively), whereas the latter is measured by the change in the ‘per capita’ effect that the donor species gives to the recipient one [7–9]. In the present paper, we used the term density dependence to refer to the latter response, which may be caused by various behavioural or physiological changes involved in the interspecific relationship. In a predator–prey relationship, for example, aggregation behaviour, cryptic or warning coloration, mimicry, switching predation, group foraging, etc., are known to exhibit density dependence on each player (e.g. [10–15]). It should also be noted that what functional forms a density-dependent effect takes may be varied with its evolutionary context that is diverse among species. For example, the predation avoidance of warning coloration improves with prey density if the prey is honestly unpalatable; otherwise, the avoidance efficiency decreases [16,17]. Thus, various forms of density dependence in interspecific interactions should coexist in a natural community.

Our aim in the present paper is to examine explicitly how density-dependence variation affects the complexity–stability relationship. The functional type of interspecific interaction is highly influential with respect to community dynamics and the likelihood of species coexistence [9,18,19]. When the impact of a species interaction increases as the density of the affected species increases (i.e. positive density dependence, hereafter PDD), harmful effects provide an advantage to less abundant species and thus stabilize interspecific relationships, whereas beneficial effects exert positive feedback by providing greater benefit to the more abundant species, thus destabilizing community dynamics. In contrast, with negative density dependence (i.e. the impact of the interaction decreases with increasing density of the recipient species, hereafter NDD), the consequences of harmful and beneficial interactions are reversed (i.e. destabilizing and stabilizing, respectively). For these reasons, several theoretical studies on the complexity–stability relationship have focused on density dependence or the functional response of species interactions [20–23]. For example, a Holling's type III functional response may yield positive complexity–stability relationships, but others may not [20] (but see [21]). Kondoh [22] demonstrated that food-web persistence improves with increasing complexity when predators show adaptive or flexible foraging in response to prey abundance. However, most of the models presented were based on an implicit assumption that density dependence occurs in either beneficial or harmful interactions, or that the density-dependent function of an interaction effect is fixed and uniform in the community (e.g. models assuming only a Holling's type I, II or III functional response). Thus, it remains unclear what role, if any, variations in density dependence play in community stability.

In this study, we present a general theory that explains how the type of (and variation in) density dependence of interspecific interactions affects community dynamics, and we provide a simple condition under which positive complexity–stability relationships emerge. To do this, we first extended the typical generalized Lotka–Volterra model to include variation in density-dependent effects, in which the *per capita* effect can be any form of density dependence for each species interaction. Then, we analytically derived a stability criterion for the model with some limited assumptions and tested the generality of the analytically derived prediction by performing numerical simulations of the local stability analysis with relaxed assumptions. Based on the analysis, we discuss the importance of density-dependence variation in the complexity–stability relationship in nature.

## 2. Methods

### (a) Mathematical model

Consider an *N*-species community, whose dynamics are driven by various types of species interactions, such as antagonistic, competitive and mutualistic relationships. Any pair of species is connected to each other with probability *C* (≤1; connectance). The population dynamics is given as2.1
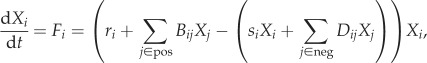
in which *X_i_*, *r_i_* and *s_i_* are the abundance, the intrinsic growth rate and the self-regulation intensity of species *i*, respectively. Parameters *B_ij_* and *D_ij_* denote the *per capita* effects of beneficial and harmful interactions that species *j* has on *i*, respectively (*i* ≠ *j*).

The *per capita* effect of an interspecific interaction may be dependent on the densities of conspecifics and allospecifics, which is described by various forms of functional response [24]. Typical community models often assume uniform or unilateral (i.e. only in beneficial or harmful effects) density-dependent functions in the community [20–23]. However, in reality, any form of functional response should coexist in the community. Here, we present a mathematical model of an ecological community in which variation in functional response is explicitly incorporated. We assume that around the equilibrium, the density dependence of *per capita* interspecific effects is approximated by an exponential function,2.2
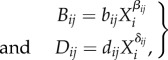
in which *b_ij_* and *d_ij_* are the slopes of the *per capita* beneficial and harmful interspecific effects, respectively (both *b_ij_* and *d_ij_* > 0). Parameters *β_ij_* and *δ_ij_* determine the functional type or density dependence of the beneficial and harmful effect that species *j* has on species *i*, respectively. *Per capita* effect on species *i* decelerates with its own density when *β_ij_* or *δ_ij_* < 0 (i.e. NDD). With an increase in the density of the recipient species *i*, the *per capita* effect with a positive density dependence (PDD) saturates as in a Holling's type II functional response when 0 < *β_ij_* or *δ_ij_* < 1 and accelerates when *β_ij_* or *δ_ij_* ≥ 1.

### (b) Analytical solution for local stability criterion

Among several indices of ecological community stability [5,6], we used local stability of feasible equilibrium, that is, the tendency of the community to return to an equilibrium when all species have positive density after small perturbations [3,25]. Such local behaviours are easily assessed by linearization of the system at an equilibrium point, which is the so-called community matrix *M* [26]. Thus, given that equation (2.1) holds that *F_i_* = 0 at an equilibrium, diagonal elements of the community matrix can be obtained as partial derivatives with respect to the density of species *i*:2.3
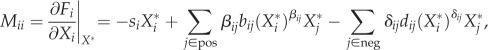
where 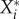 denotes the equilibrium density of species *i* (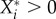). For the off-diagonal elements, *M_ij_* is set to 0 when species *j* has no effects on *i*. On the other hand, with positive and negative species effects, the off-diagonal elements become2.4
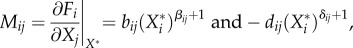
respectively.

The system is locally stable if all eigenvalues of the community matrix have negative real parts. For a random community matrix with 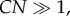 *E*(*M_ij_*) = 0 and *E*(*M_ij_M_ji_*) = 0, the stability criterion is analytically given by2.5
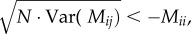
[3,25,27]. To ensure the above conditions, we here assumed some constraints in the community dynamics model with the density-dependence variation. First, for the simplicity, parameters and species abundances are set as constant (*s_i_* = *s*, *b_ij_* = *b*, *d_ij_* = *d*, *β_ij_* = *β*, *δ_ij_* = *δ* and 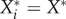). Then, positive and negative interspecific interactions are set to be of the same magnitude on average (*d* = *b*(X*)*^β^*^−*δ*^) to hold that *E*(*M_ij_*) = 0. As any pair of species is randomly connected with probability *C* in the present model, there is no statistical correlation between the off-diagonal elements of the interaction matrix (i.e. *E*(*M_ij_M_ji_*) = 0). Therefore, for large systems, the diagonal elements *M_ii_* and Var(*M_ij_*) can be approximated by2.6
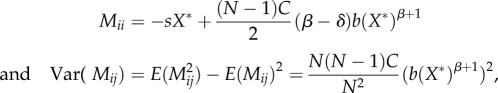
respectively (a species is expected to interact with (*N* − 1)*C* allospecifics). Substituting equation (2.6) into equation (2.5) and assuming *N* − 1 ≈ *N* for 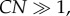 we have the stability criterion for communities with the density-dependence variation, given by2.7
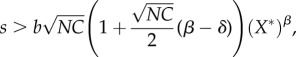
which is identical to May's stability criterion [3] without any density dependence (i.e. *s* > *b*(*NC*)^1/2^ when *β* = *δ* = 0).

### (c) Numerical simulation with relaxed assumptions

The local stability criterion obtained above was limited to the case that meets the condition *E*(*M_ij_*) = 0 and *E*(*M_ij_M_ji_*) = 0, in which parameters and equilibrium densities are constant among species, and the community matrix is constructed with random interaction. Here, to investigate the effect of the density-dependence variation under more realistic situations, we further performed numerical simulations while relaxing the assumptions. First, the self-regulation intensity *s_i_* and an equilibrium density of each species 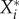 were randomly chosen from uniform distributions *U*(0, *s*_max_) and *U*(0, *X*_max_), respectively. The effects of beneficial interactions *b_ij_* and harmful interactions *d_ij_* were also chosen from uniform distributions *U*(0, *b*_max_) and *U*(0, *d*_max_), respectively.

Finally, to reflect the non-random network structure of real communities [28], we introduced interaction-type diversity into the model [27,29]. Specifically, in the simulation with the multiple interaction types, any interaction between species *i* and *j* (*i* ≠ *j*) was categorized as one of four interaction types: predator–prey, competition, mutualism and random, with the proportions *p*, *c*, *m* and 1 − (*p* + *c* + *m*), respectively. The slopes of competitive, mutualistic and random interaction effects were chosen from *U*(−1, 0), *U*(0, 1) and *U*(−1, 1), respectively. The slopes of interaction for the predator and prey were also chosen from *U*(0, 1) and *U*(−1, 0), respectively.

Based on the signs of the interaction matrix, the density-dependence parameters *β_ij_* and *δ_ij_* were randomly assigned from the uniform distributions *U*(*β*_min_, *β*_max_) and *U*(*δ*_min_, *δ*_max_), respectively. In the numerical simulations, PDD and NDD condition were defined as the mean value of the density-dependence distribution (the mean density dependence of beneficial and harmful interactions was *β*_mean_ = (*β*_min_ + *β*_max_)/2 and *δ*_mean_ = (*δ*_min_ + *δ*_max_)/2, respectively), because each interspecific interaction can be PDD and NDD depending on the distribution range. After setting parameters, the intrinsic growth rates *r_i_* were determined to hold that 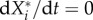. Thus, under these relaxed assumptions, off-diagonal elements of the community matrix *M_ij_* can take any value without the constraints *E*(*M_ij_*) = 0 and *E*(*M_ij_M_ji_*) = 0, and the equilibrium densities 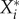 were different among species. To investigate the local stability of the community, we calculated the eigenvalues of the obtained community matrix. Stability was defined as the proportion of communities in which the largest real part of the eigenvalue becomes negative among 1000 samples.

## 3. Results

### (a) Analytical solution for local stability criterion

**Figure 1.**
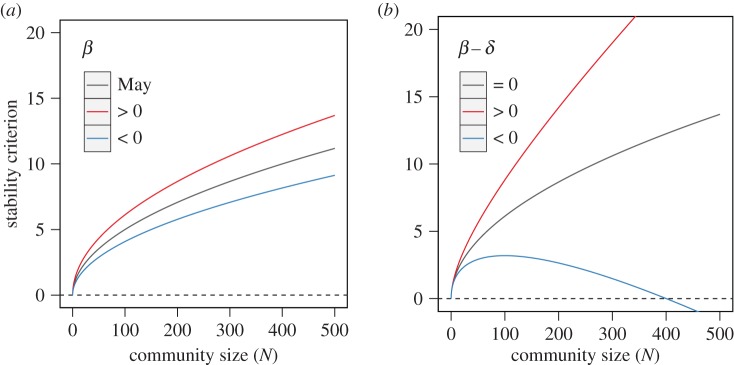
Stability criterion for a random community with density-dependence variation. The lines represent the behaviour of the right-hand side of equation (2.7) as a function of community size *N*. (*a*) Equal density dependence between beneficial and harmful interactions (i.e. *β* = *δ*; *β* = 0, 0.5 and −0.5 for grey, red and blue lines, respectively). (*b*) The effect of density-dependence variation on the stability criterion (*β* = 0.5; *δ* = 0.5, 0.4 and 0.6 for grey, red and blue lines, respectively). Other parameters: *s* = 1.0, *C* = 1.0, X* = 1.5 and *b* = 0.5.

Figure 1 shows how the minimum self-regulation intensity that is required for local stability (the right-hand side of equation (2.7)) changes with community size *N* (the connectance is fixed as *C* = 1). In communities with symmetric density dependence, the NDD condition (i.e. *β* = *δ* < 0) simply promotes local stability, whereas the PDD condition decreases stability compared with May's criterion; however, the value monotonically increases with an increase in *N*, indicating that the traditional negative relationship between complexity and stability is qualitatively unchanged in all cases (figure 1*a*). On the other hand, the stability criterion equation (2.7) demonstrates that variation in density dependence (i.e. *β* − *δ* ≠ 0) dramatically changes the complexity–stability relationship. More specifically, if *β* − *δ* > 0 the stability criterion shows an accelerated rise, whereas if *β* − *δ* < 0 the criterion shows a convex curve as a function of community size, and finally falls below the upper threshold of stability in large communities (figure 1*b*). That is, a positive complexity–stability relationship emerges whenever the density dependence of harmful species interactions is larger than that of beneficial interactions.

### (b) Local stability analysis with numerical simulations

**Figure 2.**
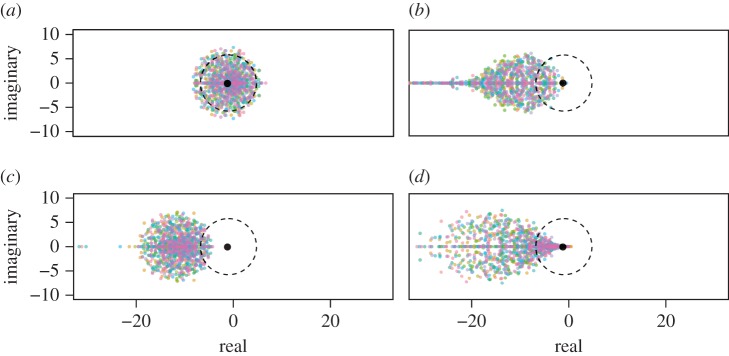
Distribution of eigenvalues for communities with density-dependence variation. Differently coloured points represent the eigenvalues of 10 random community matrices. (*a*) The case of an equal density dependence (such that both beneficial interactions *β_ij_* and harmful interactions *δ_ij_* are derived from *U*(−0.2, 0.2)). (*b*) *β_ij_* = *U*(−1.0, 0.0) and *δ_ij_* = *U*(−0.3, 0.1). (*c*) *β_ij_* = *U*(−0.4, 0.0) and *δ_ij_* = *U*(0.0, 0.4). (*d*) *β_ij_* = *U*(−0.1, 0.3) and *δ_ij_* = *U*(0.0, 1.0). The dashed ellipses represent predictions based on May's stability criterion. Other parameters: *N* = 100, *C* = 1.0, *s*_max_ = 2.0, *X*_max_ = 2.0, *b*_max_ = 1.0 and *d*_max_ = 1.0.

Figure 2 illustrates how the density-dependence variation affects the eigenvalue distribution of the random community matrix. As shown in the figure, when the degree of density dependence was equal between beneficial and harmful interaction (i.e. *β*_mean_ − *δ*_mean_ = 0), random communities have an eigenvalue distribution similar to the analytical prediction based on May's stability criterion (figure 2*a*). In contrast, the eigenvalue distribution is skewed towards negative values in the real number axis whenever the discrepancy in the density-dependence, *β*_mean_ − *δ*_mean_ < 0, exists (figure 2*b–d*). This indicates that such differences in density dependence have a stabilizing effect on ecological communities, although there are differences in the distribution among the parameter combinations (figure 2*b*–*d*).

**Figure 3.**
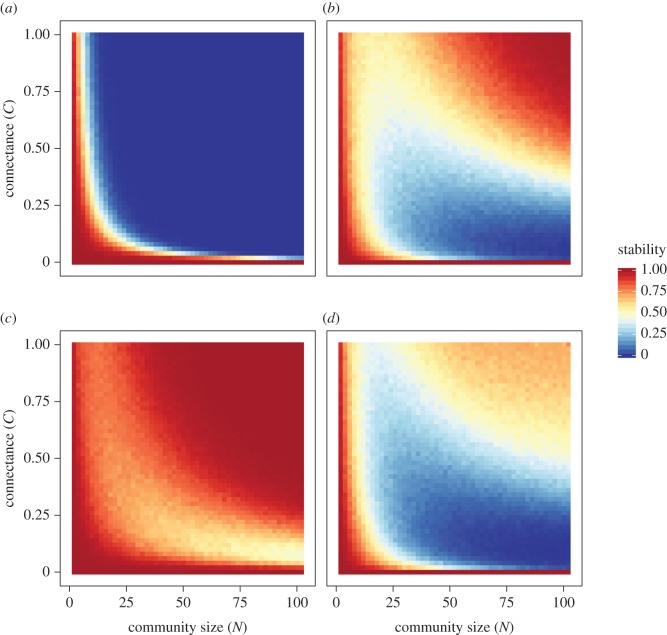
The complexity–stability relationship in random communities with or without density-dependence variation. Different colours indicate the proportions of stable communities among 1000 samples. The distributions of density dependence used were (*a*) *β_ij_* = *δ_ij_* = *U*(−0.2, 0.2) (i.e. the condition comparable to the May's stability criterion), (*b*) *β_ij_* = *U*(−1.0, 0.0) and *δ_ij_* = *U*(−0.3, 0.1), (*c*) *β_ij_* = *U*(−0.4, 0.0) and *δ_ij_* = *U*(0.0, 0.4), and (*d*) *β_ij_* = *U*(−0.1, 0.3) and *δ_ij_* = *U*(0.0, 1.0). Other parameters: *s*_max_ = 2.0, *X*_max_ = 2.0, *b*_max_ = 1.0 and *d*_max_ = 1.0.

Further numerical simulations demonstrated that the density-dependence variation dramatically affects the complexity–stability relationship in the random communities (figure 3). Specifically, whereas the complexity–stability relation corresponds to that of May's model when the degree of density dependence was equal between beneficial and harmful interactions (figure 3*a*), the positive complexity–stability relationships arise once the density-dependence parameters meet the condition that *β*_mean_ − *δ*_mean_ < 0 (figure 3*b*–*d*). For example, when both the beneficial and harmful interactions tend to be negatively density dependent (figure 3*b*), the community stability shifts from initial drop to increment as the community size and interaction connectivity increase. This indicates that the assumptions of equal parameters and equilibrium densities among species are not essential to the effect of the density-dependence variation. In particular, the stabilizing effect was highest when the distributions of beneficial and harmful interactions had NDD and PDD mean, respectively (figure 3*c*), whereas it is lowest in communities where both interactions had PDD mean (figure 3*d*). That is, unlike the analytical prediction above, the community stability pattern was quantitatively different even though the difference between mean density dependence of beneficial interactions and that of harmful interactions was held to be an equal value (*β*_mean_ − *δ*_mean_ = −0.4 in figure 3*b*–*d*).

**Figure 4.**
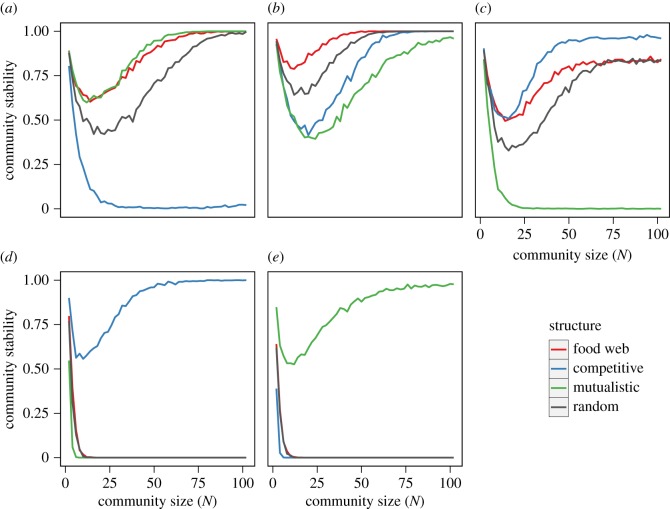
Numerically calculated stability of structured communities with density-dependence variation. Each coloured line represents the proportion of structured communities with stable equilibrium: red, blue, green and grey lines are the results of food-web-dominated communities ((*p*, *c*, *m*) = (0.4, 0.2, 0.2) for (*a*–*c*) and (0.7, 0.1, 0.1) for (*d*–*e*)), those of competition-dominated communities ((*p*, *c*, *m*) = (0.2, 0.4, 0.2) for (*a*–*c*) and (0.1, 0.7, 0.1) for (*d*–*e*)), those of mutualism-dominated communities ((*p*, *c*, *m*) = (0.2, 0.2, 0.4) for (*a*–*c*) and (0.1, 0.1, 0.7) for (*d*–*e*)) and those of random-dominated communities ((*p*, *c*, *m*) = (0.2, 0.2, 0.2) for (*a*–*c*) and (0.1, 0.1, 0.1) for (*d*–*e*)). The distributions of density dependence used were (*a*) *β_ij_* = *U*(−0.7, −0.3) and *δ_ij_* = *U*(−0.3, 0.1); (*b*) *β_ij_* = *U*(−0.4, 0.0) and *δ_ij_* = *U*(0.0, 0.4); (*c*) *β_ij_* = *U*(−0.1, 0.3) and *δ_ij_* = *U*(0.3, 0.7); (*d*) *β_ij_* = *U*(0.9, 1.3) and *δ_ij_* = *U*(0.8, 1.2); (*e*) *β_ij_* = *U*(−1.2, −0.8) and *δ_ij_* = *U*(−1.3, −0.9). Other parameters: *C* = 1.0, *s*_max_ = 2.0 and *X*_max_ = 2.0.

We further found that, with the density-dependence discrepancies, a positive complexity–stability relationship occurs even in structured networks (i.e. communities dominated by trophic, competitive and mutualistic interactions; figure 4). The results also indicated that the stabilizing effect of the density-dependence variation differs among network structures. Specifically, even though the density-dependence variation held the condition *β*_mean_ − *δ*_mean_ < 0 (figure 4*a*–*c*), competitive and mutualistic communities no longer show a positive complexity–stability relationship under the NDD distributions (i.e. both *β*_mean_ and *δ*_mean_ are negative; figure 4*a*) and the PDD distributions (both *β*_mean_ and *δ*_mean_ are positive; figure 4*c*), respectively. On the other hand, when the density-dependence variation did not hold the condition, the positive complexity relationship occurs in competitive communities with PDD mean (*β*_mean_ > *δ*_mean_ > 0; figure 4*d*) and in mutualistic communities with NDD mean (0 > *β*_mean_ > *δ*_mean_; figure 4*e*).

## 4. Discussion

We extended the traditional mathematical model of community dynamics to incorporate variations in the density dependence of interspecific interactions and revealed that variations in the density dependence of interspecific effects dramatically impact community stability and the complexity–stability relationship. Community stability is enhanced by PDD and NDD of harmful and beneficial interspecific effects, respectively. Furthermore, the classically negative complexity–stability relationship is reversed when harmful interspecific effects have larger density dependence than beneficial interspecific effects, regardless of whether the density dependence is positive or negative.

One might consider that the stabilizing effects are attributable to the combination of PDD of harmful effects and NDD of beneficial effects. That is, the PDD of negative effects and NDD of positive effects combined results in density-dependent growth such that the population receives more positives when its density is small and more negatives when its density is large. This strengthens self-regulatory mechanisms and thus stabilizes the dynamics. However, this condition does not explain all of the observed patterns, as the emergence of a positive complexity–stability relationship does not necessitate the condition of strong self-regulation. Rather, the condition under which a positive complexity–stability relationship arises is that in which harmful effects are more density dependent than beneficial effects, regardless of the signs (i.e. positive or negative) of their dependence; there is a chance for a positive complexity–stability relationship to arise even when both interspecific effects tend to be negatively or positively density dependent (figure 3*b*,*d*). This is because the stabilizing effects of species interactions (e.g. harmful interactions with PDD) may cancel out the destabilizing effects (e.g. beneficial interactions with PDD). Importantly, this cancelling effect necessarily accumulates with an increase in ecological complexity, or in the number of interacting species. Thus, only a slight difference in mean density dependence is required to yield a positive complexity–stability relationship (equation (2.7); figure 3*b*–*d*). Our additional simulations confirmed this conclusion, as the positive complexity–stability relationships occurred whenever the condition *β*_mean_ − *δ*_mean_ < 0 was met, regardless of the ranges in density-dependence distribution (electronic supplementary material, figure S1).

The present theory has a link to existing predictions on how functional response affects community stability. Nunney [20] argued that a Holling's type III functional response can yield a positive complexity–stability relationship. Specifically, with a simple approximation of diagonal (self-regulatory) elements and off-diagonal (destabilizing) elements of community matrices with a type III curve, the magnitude of self-regulatory terms in the stability criterion increases faster than off-diagonal terms with increasing connectance [20]. This prediction was questioned by Abrams & Allison [21], who provided some counterexamples using models of low-dimensional (4–10 species) systems and argued that the positive complexity–stability relationship with the type III curve is based on unrealistic assumptions [30]. However, our additional comprehensive analysis strengthens the logical basis of Nunney's argument (see the text in the electronic supplementary material). We argue that the apparent contradiction between the two studies stems from the difference in the assumed system size. Here, the point is that food-web models with functional responses, such as Holling's type I, II and III responses, can be viewed as models with density dependence for harmful interspecific effects alone (i.e. *β* = 0, *δ* ≠ 0). A type III functional response leads to an accelerating attack rate (i.e. *δ* > 0) at low prey density levels, and thus can give rise to a positive complexity–stability relationship (equation (S5) and figure S3 in electronic supplementary material). Abrams & Allison [21] would have found a positive connectance–stability relationship if they had used a model with more species (electronic supplementary material, figure S3*a*).

Density-dependence variation adds to our understanding of how interaction types and their diversity affect species coexistence. Recently, several theoretical studies have investigated the effects of mixing multiple interaction types (i.e. trophic, competitive and mutualistic interactions) on the complexity–stability relationship [27,29,31–33]. However, as these models assumed specific conditions (e.g. uniform density dependence or constant interaction effort), the general effect of multiple interaction types remains less well understood. Using more general modelling, the present study explicitly reveals that whether or not the positive complexity–stability relationship arises is synergistically determined by the interaction type and the variation in density dependence (figure 4). For example, when harmful interactions have larger density dependence than beneficial ones and the both of these have PDD distributions, increasing complexity destabilizes mutualistic communities while stabilizing the others (figure 4*c*). On the other hand, when *β*_mean_ − *δ*_mean_ < 0 and both of these have NDD distributions, the positive complexity–stability relationship disappears only in competitive communities (figure 4*a*). In extreme cases, the stabilizing effect of increasing complexity arises in competitive or mutualistic communities even when *β*_mean_ − *δ*_mean_ > 0 (figure 4*d*,*e*). Note that the perfect competition and mutualism significantly decreased the community stability (electronic supplementary material, figure S2). In particular, the positive complexity–stability relations arise in perfectly competitive networks when competitions have a moderate PDD mean (electronic supplementary material, figure S2*b*,*c*) but do not in mutualistic networks when mutualisms have a moderate NDD mean (electronic supplementary material, figure S2*a*,*b*). This is caused by the fact that the positive feedback of mutualistic interactions is so strong that moderate NDDs cannot cancel out the destabilizing effect. Thus, whether or not increasing complexity stabilizes ecological community depends on both interaction-type mixing and density-dependence variation.

The present theory predicts that complex communities can be stable if harmful interspecific effects have larger density dependence than beneficial ones. For example, a food web shows the complexity–stability relationship if prey species suffer from predation attacks accelerating with their own density (yielding PDD in harmful effects) and/or predator species interfere with each other in terms of consuming prey (yielding NDD in beneficial effects). However, there is virtually no evidence that this condition actually holds true in nature. While much effort has been made to describe the pattern of the functional response of predator attack rate (i.e. density dependence of harmful effects on prey species; e.g. [34–38]), we know little about the density dependence of the beneficial effects that a predator species receives from its prey [39]. We have even less information for other relationships, such as mutualism [40,41]. The theory of evolutionary biology may help to compensate for this information shortage. For example, optimal foraging theory predicts that preferential attacks on abundant and palatable prey are favourable to predators, yielding predation effects with PDD on prey density [42,43]. This may explain the frequent observations of adaptive foraging behaviour across diverse taxa [37,44]. Some community theories also assume saturating benefits of mutualism [40,41], although the evolutionary mechanism underlying such functional responses are unknown. However, as mutualism is often compared with consumer–resource relationships [41,45], the optimal foraging logic might be applicable, weakening the PDDs in mutualistic interactions. Thus, in light of the density-dependence variation, unifying predictions from evolutionary theory and empirical surveys of functional responses may advance our understanding of ecological complexity–stability in the real world.

## Supplementary Material

Electronic Supplementary Material

## Data accessibility

This article has no additional data.
