# Supplementary material for: Density-dependent interspecific interactions and the complexity–stability relationship
Source: Proc Biol Sci. 2018 May 23;285(1879):20180698. doi: 10.1098/rspb.2018.0698 (PMC5998089; doi:10.1098/rspb.2018.0698)
Supplement: Electronic Supplementary Material [file rspb20180698supp1.pdf]

Electronic Supplementary Material

Analytical stability criterion for the classical functional response

Incorporating the classical functional response into equation 1 in the main text, we obtain a familiar species dynamics equation:

$$F_i = \left(r_i + \sum_{j \in \text{prey}} \frac{b_{ij} X_j^n}{1 + h_{ij} X_j^n} - \left(s_i X_i + \sum_{j \in \text{predator}} \frac{d_{ij} X_i^{n-1}}{1 + h_{ji} X_i^n} X_j \right) \right) X_i, \qquad \text{(S1)}$$

in which the parameters n and h_{ij} represent the type of functional response (i.e., Holling's type I, II and III) and the saturating parameter in consuming prey j, respectively. Then, given that equation S1 holds that $F_i = 0$ at the equilibrium, diagonal elements of the community matrix can be obtained as:

$$M_{ii} = \left.\frac{\partial F_i}{\partial X_i}\right|_{X^*} = -s_i X_i^* - \sum_{j \in \text{predator}} \frac{\left(n - 1 - h_{ji}(X_i^*)^n\right) d_{ij} (X_i^*)^{n-1}}{\left(1 + h_{ji}(X_i^*)^n\right)^2} X_j^*. \qquad \text{(S2)}$$

The off-diagonal elements with predator and prey species become:

$$M_{ij} = \left.\frac{\partial F_i}{\partial X_j}\right|_{X^*} = \frac{n b_{ij} \left(X_j^*\right)^{n-1}}{\left(1 + h_{ij}\left(X_j^*\right)^n\right)^2} X_i^* \text{ and } -\frac{d_{ij}(X_i^*)^n}{1 + h_{ji}(X_i^*)^n}, \qquad \text{(S3)}$$

respectively, if an interaction between species i and j occurs (otherwise M_{ij} are set to 0).

To obtain an analytical stability criterion, we consider a random community with $CN >> 1$ and $E(M_{ij}M_{ji}) = 0$; the parameters are set as constant ($s_i = s$, $b_{ij} = b$, $d_{ij} = d$, $h_{ij} = h$ and $X_i^* = X^*$) and the condition $b = d(1 + h(X^*)^n)/n$ holds to ensure $E(M_{ij}) = 0$. Under these assumptions, as a species is expected to interact with $(N - 1)C/2$ predators, the diagonal elements M_{ii} and $Var(M_{ij})$ are approximated by:

$$M_{ii} = -sX^* - \frac{(N-1)C}{2} \cdot \frac{(n - 1 - h(X^*)^n)}{(1 + h(X^*)^n)^2} d(X^*)^n, \text{ and} \qquad \text{(S4)}$$

$$Var(M_{ij}) = E(M_{ij}^2) - E(M_{ij})^2 = \frac{N(N-1)C}{N^2}\left(\frac{d(X^*)^n}{1+h(X^*)^n}\right)^2,$$

respectively. Substituting equation S4 into equation 5 in the main text, and assuming $N - 1 \approx N$ to meet the condition that $CN >> 1$, we obtain the stability criterion for communities with the classical functional response

$$s > \sqrt{NC}\left(1 - \frac{\sqrt{NC}}{2} \cdot \frac{n-1-h(X^*)^n}{1+h(X^*)^n}\right)\frac{d(X^*)^{n-1}}{1+h(X^*)^n}, \quad \text{(S5)}$$

which corresponds to May' stability criterion [3] under the type I functional response with no handling times (i.e., $n = 1$ and $h = 0$). As seen in the right-hand side of equation S5, complexity always exerts a negative effect on community stability under the type II functional response ($n = 1$, $h > 0$). On the other hand, under the type III functional response ($n = 2$, $h > 0$), the positive complexity-stability relationship occurs if the parameters hold the condition $1 - h(X^*)^n > 0$ (this condition is consistent with the heuristic prediction of Nunney [20]).

Then, to test the prediction while relaxing the above assumptions, we performed numerical simulations using the following procedure. First, the self-regulation s_i, equilibrium density of each species X_i^*, effect of beneficial interactions b_{ij} and that of harmful interactions d_{ij} were determined in the manner described in the main text. The saturating parameter h_{ij} was chosen from a uniform distribution $U(0, h_{\max})$. We set the functional response parameter n constant and uniform distribution as either 1 (for the type II curve) or 2 (for the type III curve). After setting parameters, the intrinsic growth rate r_i of each species was determined to hold to $F_i = 0$. To investigate local stability of the random community, we calculated the eigenvalues of the obtained community matrix. Stability is defined as the proportion of communities in which the largest real part of the eigenvalue becomes negative among 1,000 samples. The results are summarised in figure S3. As predicted, type II curves yield strongly negative complexity stability relationships in random communities (figure S3*a*). For type III curves,

the positive connectance-stability relationship arose in the large communities with the low-density level (figure S3*b*) but the connectance destabilises those with the high-density level (figure S3*c*).

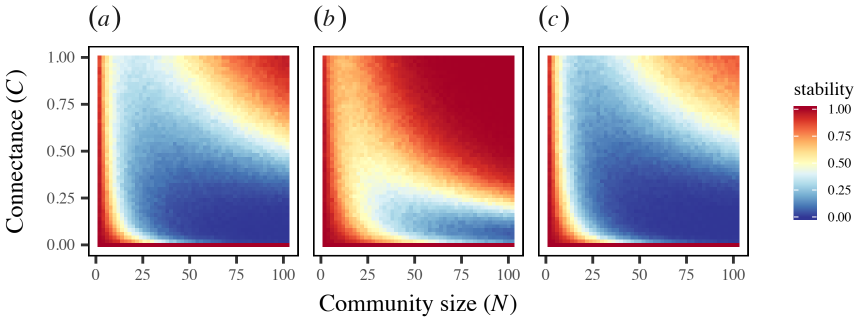

Figure S1. The complexity-stability relationship in random communities with density-dependence variation. Different colours indicate the proportions of stable communities among 1,000 samples. The density-dependence parameters used here were determined so as to preserve the mean density-dependence in figure 3*b*–*c* but the ranges of density-dependence were larger than those of figure 3 as (*a*): β_{ij} = *U*(-1.25, 0.25) and δ_{ij} = *U*(–0.55, 0.35); (*b*): β_{ij} = *U*(-0.65, 0.25) and δ_{ij} = *U*(–0.25, 0.65); (*c*) β_{ij} = *U*(-0.35, 0.55) and δ_{ij} = *U*(–0.25, 1.25). Other parameters: $s_{\max}$ = 2.00, $X_{\max}$ = 2.00, $b_{\max}$ = 1.00 and $d_{\max}$ = 1.00.

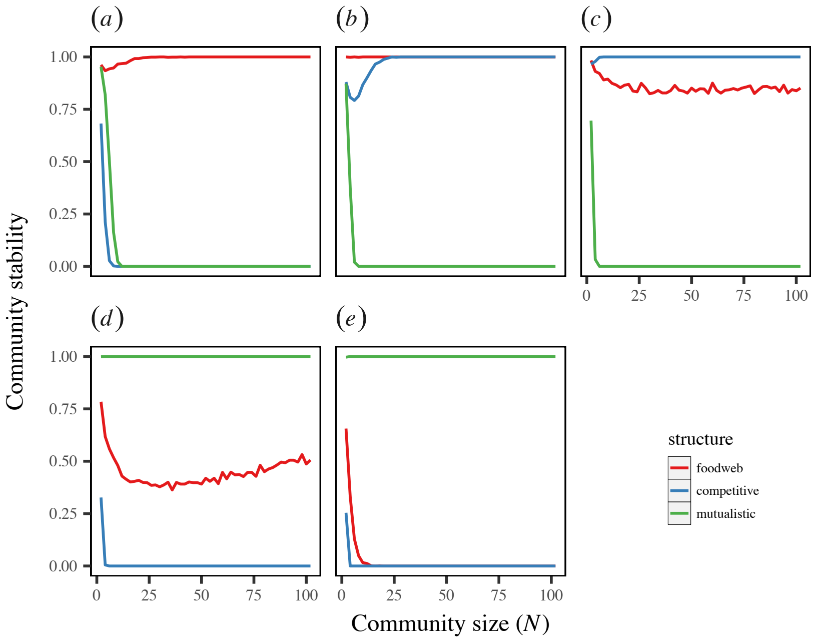

Figure S2. Numerically calculated stability of structured communities with density-dependence variation. Each coloured line represents the proportion of structured communities with stable equilibrium; red, blue and green lines are the results of perfect food-webs, perfectly competitive networks and perfectly mutualistic networks, respectively ((p, c, m) = (1.00, 0.00, 0.00), (0.00, 1.00, 0.00) and (0.00, 0.00, 1.00), respectively). The distributions of density dependence used were (*a*): $\beta_{ij} = U(-0.70, -0.30)$ and $\delta_{ij} = U(-0.30, 0.10)$; (*b*) : $\beta_{ij} = U(-0.40, 0.00)$ and $\delta_{ij} = U(0.00, 0.40)$; (*c*): $\beta_{ij} = U(-0.10, 0.30)$ and $\delta_{ij} = U(0.30, 0.70)$; (*d*): $\beta_{ij} = U(-1.20, -0.80)$ and $\delta_{ij} = U(-0.80, -0.40)$; (*e*): $\beta_{ij} = U(-1.20, -0.80)$ and $\delta_{ij} = U(-1.30, -0.90)$. Other parameters: $C = 1.00$, $s_{max} = 2.00$ and $X_{max} = 2.00$.

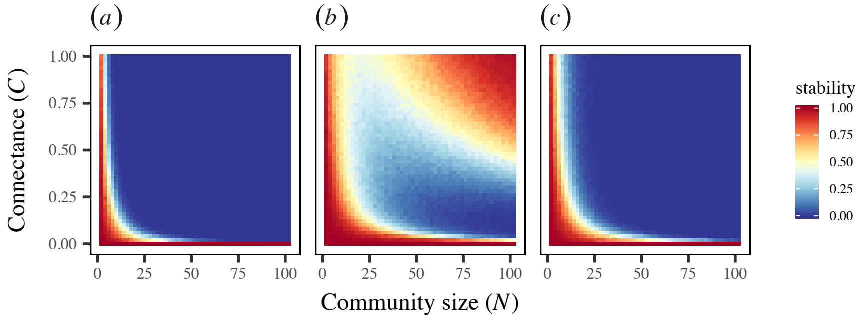

Figure S3. The complexity-stability relationship in random communities with a classical functional response. Different colours indicate the proportion of stable communities among 1,000 samples. (*a*): (X^*, n, h) = (1.00, 1.00, 1.00), (*b*): (X^*, n, h) = (1.25, 2.00, 1.00), (*c*): (X^*, n, h) = (2.50, 2.00, 1.00). Other parameters: $s = 1.00$, $b = 1.00$, $d = 1.00$.
